# Right atrial and right ventricular strain in patients with acute decompensated heart failure: a pilot study

**DOI:** 10.3389/fcvm.2025.1621473

**Published:** 2025-09-26

**Authors:** Jakub Jurica, Martin Jozef Péč, Marek Cingel, Tomáš Bolek, Boris Focko, Marianna Barbierik Vachalcová, Marián Mokáň, Matej Samoš

**Affiliations:** ^1^ Department of Internal Medicine I, University Hospital Martin, Jessenius Faculty of Medicine, Comenius University in Bratislava, Martin, Slovakia; ^2^ Department of Cardiology I, Faculty of Medicine, P.J. Šafárik University in Košice and East-Slovakian Institute of Heart and Vessel Diseases (VÚSCH, a.s.) in Košice, Košice, Slovakia; ^3^ Division of Acute and Interventional Cardiology, Department of Cardiology and Angiology II, Mid-Slovakian Institute of Heart and Vessel Diseases (SÚSCCH, a.s.) in Banská Bystrica, Banská Bystrica, Slovakia

**Keywords:** right ventricular strain, right atrial strain, speckle-tracking echocardiography, acute decompensated heart failure, right ventricular dysfunction

## Abstract

**Aims:**

The aim of this study was to compare the right atrial (RAS: reservoir-R, conduit-CD, contraction-CT) and right ventricular strain (global longitudinal-RV-GS, free wall strain-RV-FWS) between patients with acute decompensation of chronic heart failure (ADHF) and a control group.

**Methods:**

This study enrolled eighteen patients admitted to our ward for ADHF. Transthoracic echocardiography (TTE) with two-dimensional speckle tracking analysis (2D ST) was performed in each patient. The cut-off value of ≤40% was used to distinguish HF with reduced (HFrEF) from HF with preserved ejection fraction (HFpEF). The control group consisted of eighteen healthy individuals with no known history of cardiovascular disease. HF patients were followed for 6-months for HF-related adverse events (cardiovascular death or HF-related hospitalisation).

**Results:**

We found that RV-GS and RV-FWS were significantly lower in ADHF in comparison with the control group (RV-GS: −15.7 ± 3.32% vs. −22.6 ± 2.26%, *p <* 0.001; RV-FWS: −19.2 ± 4.7% vs. −25.9 ± 2.54%, *p <* 0.001). There was no significant difference in RV-GS and RV-FWS between the HFrEF and HFpEF subgroups. Additionally, R-RAS, CD-RAS and CT-RAS were significantly changed in HF patients compared to controls (R-RAS: 10.1 ± 5.5% vs. 42.5 ± 11.8%, *p* < 0.001; CD-RAS −8.1 ± 4.5% vs. −27.5 ± 9.3%, *p* < 0.001; CT-RAS: −2.1 ± 1.0% vs. −14.3 ± 6.1%, *p* < 0.001), and predicted major cardiovascular event in a 6-month follow-up period.

**Conclusion:**

Our study demonstrated a significant difference in RAS, RV-GS and RV-FWS in patients with acute decompensation of HF in comparison with the control group, with no significant difference between the HFrEF and HFpEF subgroups. RAS predicted adverse events in a 6-month follow-up period.

## 1 Introduction

Two-dimensional speckle tracking analysis (2D ST) represents an echocardiographic method that provides an assessment of myocardial deformation, and it can be used as a diagnostic tool to determine the function of any heart chamber. 2D ST is based on detecting a certain object in an echocardiographic image and analysing its deformation (the local changes in shape and size within the myocardial tissue itself, like thickening, thinning, or twisting) over the course of a heart cycle. The percentage change in the position of such an object is expressed as strain, which stands for the unit of 2D ST. 2D ST can be applied to any heart chamber. In current clinical practice, it is predominantly used to determine the systolic function of the left ventricle (LV) and left atrium (LA). LV function is usually determined as global longitudinal strain of LV (LV-GLS). The function of the left atrium is expressed as left atrial reservoir strain (R-LAS) (1). However, much less is known about the clinical relevance and utility of determining deformation parameters of the right ventricle, particularly the right ventricular global strain (RV-GS) and the right ventricular free wall strain (RV-FWS). For a long time, the standard echocardiographic measurements for evaluation of RV function were: tricuspid annular plane systolic excursion (TAPSE) by M-mode, the movement of the tricuspid annulus by pulsed-wave tissue Doppler imaging (TDI) = systolic excursion velocity and fractional area change of the right ventricle (2). RV-GS and RV-FWS provide measurements of the longitudinal strain of RV, i.e., the shortening (deformation) of RV's wall during systole and diastole, where RV-GS evaluates deformation of the entire wall of RV (free wall & interventricular septum) while RV-FWS focuses on deformation of the free wall of RV itself. RV deformation parameters seem to be more sensitive in determining the systolic function of RV compared to the conventional parameters presented above. Moreover, RV strain parameters can potentially assess myocardial contractility less-dependently of volume/pressure load compared to traditional parameters like TAPSE, making it more robust or sensitive for the determination of RV systolic function, as well as these values may be used as a possible predictors for various cardiovascular diseases (2). While not completely independent of load, its improved accuracy allows for better detection of subtle or subclinical RV dysfunction, particularly in conditions like pulmonary hypertension, which significantly affect RV afterload. Nevertheless, RV longitudinal strain is not completely independent on RV preload; a reduction in preload leads to decreased strain, while increased preload can lead to a larger contribution of longitudinal strain to systolic function. Therefore, interpreting RV longitudinal strain with caution is necessary, considering the patient's loading conditions, as changes in preload can alter these strain measurements.

Recently, reduced RV-FWS was associated with impaired prognosis in patients with acute decompensated (AD) heart failure (HF), especially in those with reduced ejection fraction due to non-ischemic aetiology and in patients with arrhythmogenic right ventricular cardiomyopathy (3–6); however the available data so far are very limited, especially in patients with HF with preserved left ventricular ejection fraction (HFpEF). Furthermore, there are minimal data about the predictive value of impaired right atrial strain (RAS) or right atrial cardiopathy in patients with AD HF and other cardiovascular diseases (CVD). Although it seems that the assessment of right atrial function can provide useful prognostic information in patients with paediatric pulmonary hypertension (7), and that reduced RAS can predict the development of kidney dysfunction in patients with tricuspid regurgitation or AD HF (8, 9), there are very limited data for prognostic value of RAS with regard to HF-associated adverse events (cardiovascular death, HF-related hospitalizations). Therefore, the prognostic value of RAS in AD HF needs to be studied more extensively.

Since there is a lack of information in the literature regarding the use of 2D-ST assessment of deformation parameters of the right atria and right ventricle and its role in patients with AD HF, the aim of this study was to assess RAS (reservoir = R-RAS, conduit = CD-RAS, contraction = CT-RAS), RV-GS and RV-FWS in patients with AD HF, compare it with individuals without any cardiovascular disease and possibly evaluate its role as a prognostic factor in predicting the risk of re-hospitalisation of patients for HF or mortality due to HF.

## 2 Methods

### 2.1 Study design

We performed a pilot, prospective, observational study. The study protocol underwent revision and approval by the local ethics committee. This study was conducted during the period from the 1st of January 2023 to the 31st of May 2023. During the study period, consecutive patients admitted to standard wards or the intensive care unit at the Department of Internal Medicine I, Teaching Hospital in Martin for AD chronic HF were enrolled in the patient group, provided they met the following inclusion criteria:

History of chronic heart failure as defined by the ESC diagnostic criteria (10);
Presence of HF signs and symptoms severe enough to seek urgent medical aid (not manageable with short-term ambulatory intravenous diuretic therapy);
NT-proNBP levels ≥300 ng/L at admission;
Adequate imaging quality on echocardiography enabling the performance of 2D ST analysis;
Absence of end-stage kidney or liver disease, absence of a recent (last 6 months) or disabling stroke or active malignancy.
A written informed consent with study participation was obtained after enrolment.
All the methods used in this study were performed in accordance with the relevant guidelines and regulations.

All the enrolled patients underwent a study echocardiographic examination for 2D ST analysis after achieving decongestion. Decongestion was defined as: the patient should have no detectable clinical signs/symptoms of congestion, on lung ultrasound, no more than 1 B-line per intercostal space should be present, the patients should have a normal diameter of inferior vena cava with inspiratory collapse, and there should be a significant decrease in NT-proBNP levels.

During the hospital stay, all patients received intravenous loop diuretic therapy. Those with systolic blood pressure ≥110 mmHg also received intravenous vasodilators; patients who presented with hypotension and/or tissue hypoperfusion on admission received intravenous inotropic therapy as well (dobutamine). The administration of intravenous digoxin and other heart failure therapies (including chronic therapy) was left to the discretion of the attending physician. After decongestion, patients were switched to oral loop diuretics with titration to effect and chronic HF therapy was up-titrated (see Section 5). There was no standardized protocol on diuretic therapy (both bolus doses or continual administration were allowed, the initial dose of loop diuretics matched at least the dose taken by the patient chronically, but the discretion on drug dosing and the way of administration—boluses vs. continuous infusion, and diuretic therapy intensification by combined diuretic therapy was left to attending physician).

As part of the final analysis, the patients were divided into 2 sub-groups according to the value of LV EF using a cut-off value of ≤40% to distinguish heart failure with reduced ejection fraction (HFrEF) from heart failure with preserved ejection fraction (HFpEF).

At the same time, a control group of healthy individuals was created. Individuals were eligible for enrolment in the control group if they met the following criteria: they should have no known prior history of any CVD, and no medication possibly affecting the CV system, they should have no family history of CVD at the age ≤ 65 years, their NT-proBNP levels should be normal, they should have normal sinus rhythm with no abnormalities on a 12-lead ECG, and a normal echocardiographic finding (no signs of structural/functional abnormalities).

All controls signed a written informed consent to take part in the study and underwent an echocardiographic examination for the assessment of 2D ST.

When assessing the RV deformation parameters as possible predictors for re-hospitalisation and mortality due to HF, data about the patient group were checked in the hospital virtual records and each patient was analysed for rehospitalisation and mortality due to HF as well as mortality regardless of the cause in the period of 3 and 6 months after index hospitalisation. HF-related adverse events were defined as death from cardiovascular causes or un-planned HF-related hospitalization with the need for intravenous diuretic therapy administration.

### 2.2 Echocardiography and 2D ST analysis

Echocardiography was performed by two experienced examinators (T.B. and M.S), who were blinded to the patients' clinical data and outcomes at the time of examination. Standard images in standard views were acquired and measured according to the recommendations of the American Society of Echocardiography and European Association of Cardiovascular Imaging (11, 12). All transthoracic echocardiography and speckle-tracking strain imaging were performed using a single ultrasound machine (VividR E95, GE Medical Systems, Milwaukee, WI, USA) with a 1.4–4.6 Hz transducer.

Speckle-tracking analysis of the RV deformation was conducted off-line by an independent experienced examinator, who was blinded to the patients' clinical data and outcomes (M.B.). All parameters were averaged over a minimum of 3 cardiac cycles. The RV endocardial border was automatically traced using a commercially available software (AFI RV GE Medical Systems, Milwaukee, WI, USA) and was manually adjusted by the performing physician. A heart cycle with End-diastole and End-systole was defined by the opening and closure of the pulmonary valve. RV-GS and RV-FWS values were acquired from the standard A4C view. Abnormal (reduced) RV-GS was defined as <−20%, and abnormal RV-FWS was defined as <−25% (11).

**Figure 1:**
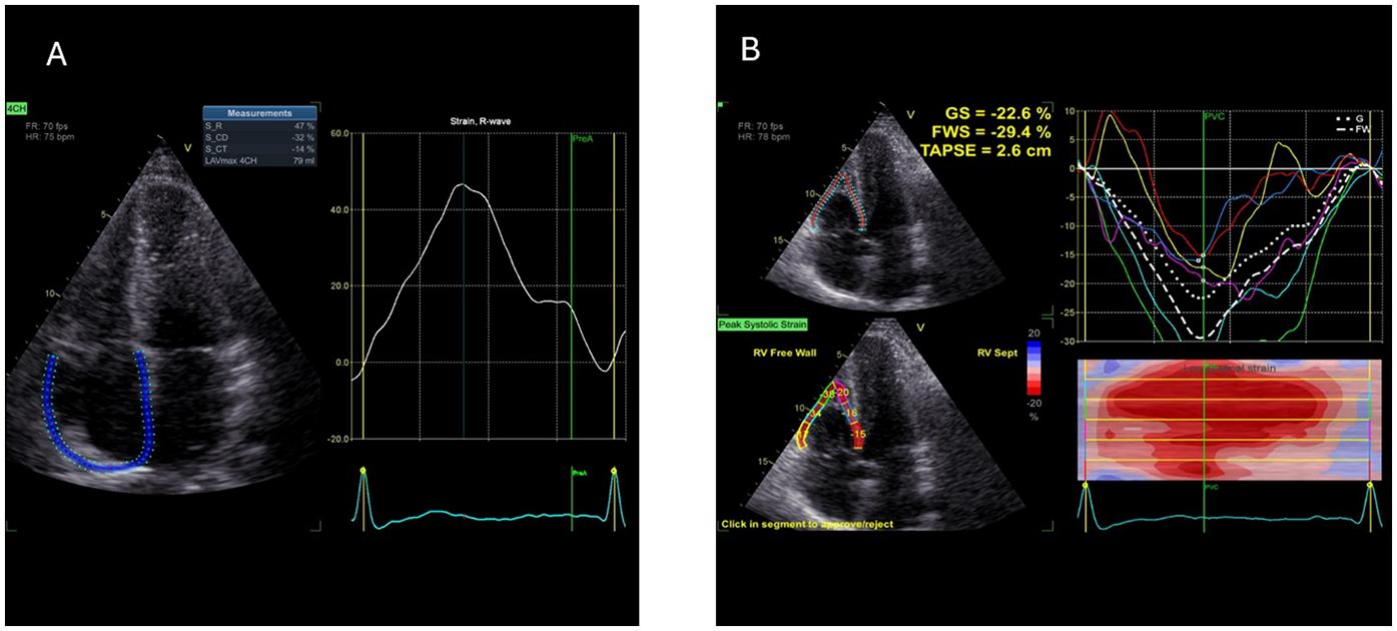
Example of 2D speckle tracking examination of right atrial **(A)** and right ventricular **(B)** strain—picture taken from control sample (2D ST, 2-dimentional speckle tracking; CD, conduit; CT, contraction; GS, global strain; FWS, free wall strain, R, reservoir; TAPSE, tricuspid anulus plane excursion).

For RAS analysis, as there was no dedicated software at the time of the study period available, a left-atrial strain dedicated commercially available software (AFI LA GE Medical Systems, Milwaukee, WI, USA) was used (off-label). Right atrial border was semi-automatically traced by the examiner: right atrial free wall was traced as septal and septal wall was traced as left atrial free wall (Figure 1), contours of right atria were than adjusted manually by the performing examiner, and then RAS was automatically assessed by the software. As mentioned, R-RAS, CD-RAS and CT-RAS were analysed.

In addition to 2D-STE analysis, standard 2D echocardiography for assessment of RA and RV structure and function was also performed. The following parameters were analysed: area of the right atrium at end-systole (RA area) in cm^2^, right ventricular basal diameter at end-diastole from apical 4-chamber view (RVD) in mm, tricuspid annular plane systolic excursion (TAPSE) in mm and tricuspid lateral annular systolic excursion velocity measured by tissue doppler imaging (Sˈ) in cm/second (s).

### 2.3 Statistical analysis

In the initial phase of our statistical analysis, the Shapiro–Wilk test was conducted to check all continuous data for normality. Based on the results of this analysis, continuous variables were reported as means ± standard deviations (for data with normal distribution) or medians and interquartile ranges (for data with asymmetrical distribution). Categorical variables were reported as number of cases (*N*) or % of cases. Differences in continuous variables between the studied groups were checked using the Student *t*-test (for normal distribution) or *u*-test (for asymmetrical one). Differences in categorical variables were assessed with the chi-squared test. Statistical analysis was performed with statistical software Statistica version 5.0 (StatSoft, Tula, OK, USA).

## 3 Results

### 3.1 Patients and controls

Throughout the study period, 18 patients with acute decompensation of chronic heart failure met the study criteria for inclusion (patient group), and the same number of individuals were enrolled to the control group. The basic demographics of the patients and controls are summarised in Table 1. The HFrEF and HFpEF subgroups consisted of six and twelve patients, respectively. Looking on the HF aetiology, in patients with HFrEF, HF developed due to myocardial ischemia (post-myocardial infarction dysfunction or chronic coronary syndrome) in 66.7% of patients and due to non-ischemic myocardial diseases (dilated cardiomyopathy or valvular disease) in 33.3% of patients. In patients with HFpEF, the ischemic HF aetiology was present in 25% of patients, the non-ischemic (hypertensive heart disease or valvular heart disease) in 58.3% of patients, and in 16.7% of patients the aetiology of HF was indeterminate.

**Table 1:** Basic review of selected laboratory parameters, comorbidities, and heart failure therapy in the HFrEF and HFpEF subgroups of patients with acute decompensation of chronic heart failure and in a control group.

Parameter	HFrEF subgroup	HFpEF subgroup	Control group
Number of patients (men/women)	6 (5/1)	12 (7/5)	18 (12/6)
Age	71 (56–78)	77 (64–88)	32 (20–54)
Beta-blockers at admission/upon release (%)	83.3/100	75/75	0
ACE inhibitors, AT1RB, ARNI at admission/upon release (%)	33.3/66.7	75/75	0
MRA at admission/upon release (%)	83.3/100	25/75	0
SGLT2i at admission/upon release (%)	16.7/16.7	0/0	0
CRT at admission/upon release (%)	0/0	0/0	0
Digoxin at admission/upon release (%)	33.3/33.3	30/30	0
Loop diuretics at admission/upon release (%)	83.3/100	50/100	0
BMI (kg/m^2^)	28 ± 5.4	27.3 ± 4.3	24.2 ± 3.8
Serum creatinine (µmol/L)	100.7 ± 35.3	129.4 ± 102.8	0
Calculated GFR–Cockcroft Gault (ml/min/1.73 m^2^)	67.5 ± 23.2	56.8 ± 27.8	0
ALT (µkat/L)	0.47 ± 0.28	0.38 ± 0.21	0
AST (µkat/L)	0.61 ± 0.30	0.55 ± 0.22	0
NT-proBNP (pg/ml)	7,153.1 ± 6,622.3	7,929.1 ± 5,427.4	0
Hemoglobin (g/L)	130.6 ± 15.9	113.1 ± 9.6	0
Total Serum Protein (g/L)	60.2 ± 15.8	63.6 ± 7.1	0
EF LV (%)	30.5 ± 9.2	53.1 ± 8.8	0
Etiology of heart failure (%) (ischemic/non-ischemic/indeterminate)	66.7/33.3/0	25/58.3/16.7	N/A
Myocardial revascularization (%)	50	30	0
History of MI (%)	66.7	30	0
Atrial fibrillation (%)	50	66.7	0
Valve disease—moderate to severe (%)	83.3	58.3	0

When comparing the parameters of patients with the control group, the controls were younger, as expected, they had lower levels NT-proBNP and serum creatinine, but there was no major difference in their body composition (body mass index = BMI). As defined by study inclusion/exclusion criteria, there was a significant difference in pharmacological therapy (as no cardiovascular pharmacotherapy should be administered in controls).

Regarding the differences between the HFrEF and HFpEF subgroups, there were no differences observed in age, liver function, NT-proBNP levels, estimated renal function or prevalence of atrial fibrillation. In addition, the HFrEF patients tended to be more frequently treated with mineralocorticoid receptor antagonists as well as sodium-glucose cotransporter-2 inhibitors at admission. However, no major difference was found in the prevalence of betablockers, loop diuretics and digoxin at admission and upon release (Table 1). The differences in RV 2D echocardiographic data between patients with HFrEF and HFpEF are reported in Table 2.

**Table 2:** Comparison of conventional 2-dimentional echocardiographic parameters of the right ventricle and right atrium between the HFrEF and HFpEF subgroups.

Parameter	HFrEF subgroup	HFpEF subgroup
TAPSE (mm)	15.0 ± 3.3	17.1 ± 3.9
S’ (cm/s)	8.5 ± 1.0	9.9 ± 2.7
RVD (mm)	45.5 ± 5.8	40.8 ± 8.5
RA area (cm^2^)	25.0 ± 8.2	22.6 ± 6.2

HFrEF, heart failure with reduced ejection fraction; HFpEF, heart failure with preserved ejection fraction; TAPSE, tricuspid annular plane systolic excursion; S’, systolic excursion velocity by TDI; RVD, right ventricular basal diameter at end-diastole; RA area, area of the right atrium at end-systole.

During the 6 months of clinical follow up, 8 ADHF patients had a HF-related adverse event, while 10 patients remained free of these events. The demographic differences between those patients who experienced and adverse event and those who did not are shown in Table 3.

**Table 3:** Basic review of selected laboratory parameters, comorbidities, and heart failure therapy in the HF patients with and without adverse events in a 6-month follow-up.

Parameter	With an adverse event	Without an adverse event
Number of patients (men/women)	10 (9/1)	8 (3/5)
Age	73 (56–82)	81 (64–88)
Beta-blockers at admission/upon release (%)	80/70	75/100
ACE inhibitors, AT1RB, ARNI at admission/upon release (%)	50/60	75/87.5
MRA at admission/upon release (%)	70/100	12.5/62.5
SGLT2i at admission/upon release (%)	10/10	0/0
CRT at admission/upon release (%)	0/0	0/0
Digoxin at admission/upon release (%)	30/30	25/25
Loop diuretics at admission/upon release (%)	90/100	25/100
BMI (kg/m^2^)	27.5 ± 2.4	27.2 ± 4.6
Serum creatinine (µmol/L)	109.9 ± 47.8	132.3 ± 127.3
Calculated GFR–Cockcroft Gault (ml/min/1.73 m^2^)	62.6 ± 27.7	57.6 ± 27.8
ALT (µkat/L)	0.42 ± 0.29	0.39 ± 0.17
AST (µkat/L)	0.57 ± 0.25	0.56 ± 0.26
NT-proBNP (pg/ml)	6,243.7 ± 5,415.1	9,453.9 ± 6,647.4
Hemoglobin (g/L)	119.4 ± 18.4	118.4 ± 8.8
Total Serum Protein (g/L)	62.3 ± 13.6	62.8 ± 5.9
EF LV (%)	41.8 ± 14.5	50.3 ± 15.5
Myocardial revascularization (%)	50	12.5
History of MI (%)	60	12.5
Atrial fibrillation (%)	70	50
Valve disease—moderate to severe (%)	60	75

HFrEF, heart failure with reduced ejection fraction; HFpEF, heart failure with preserved ejection fraction; ACE, angiotensin converting enzyme; AT1RB, angiotensin 1 receptor blocker; ARNI, angiotensin receptor/neprilysin inhibitor; MRA, mineralocorticoid receptor antagonist; SGLT2i, sodium-glucose cotransporter-2 inhibitor; CRT, cardiac resynchronization therapy; BMI, body mass index; GFR, glomerular filtration rate; ALT, alanine aminotransferase; AST, aspartate aminotransferase; NT-proBNP, N-terminal prohormone of brain natriuretic peptide; EF LV, ejection fraction of left ventricle; MI, myocardial infarction.

### 3.2 Right ventricular 2D ST analysis

**Figure 2:**
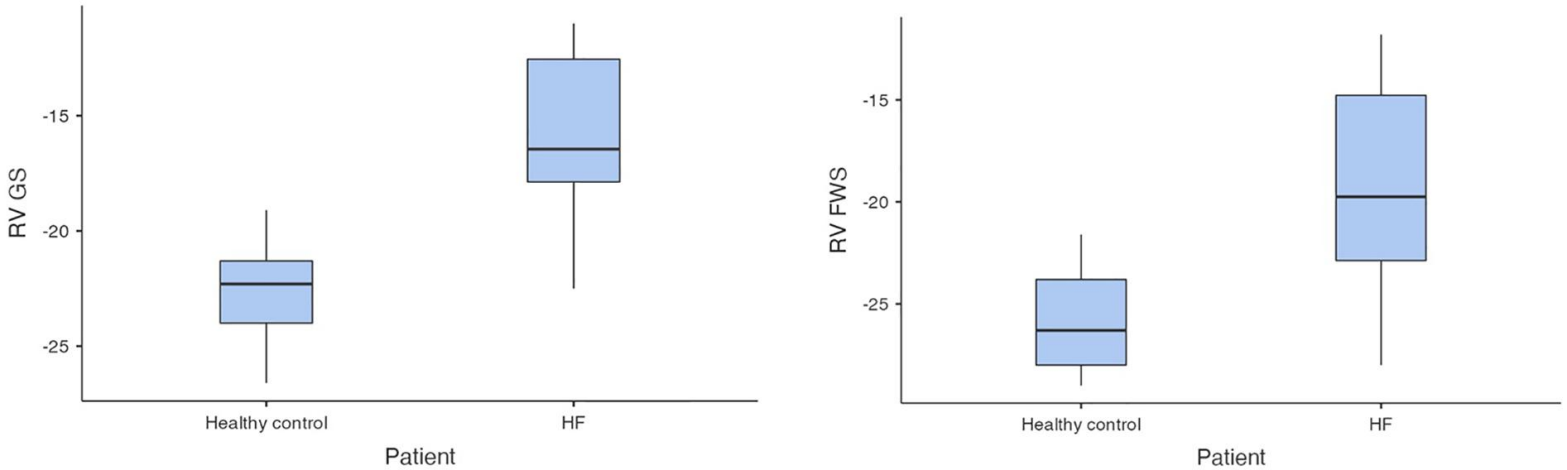
Comparison of RV-GS and RV-FWS between patients with HF and control group (HF, heart failure; RV-GS, right ventricular global strain; RV-FWS, right ventricular free wall strain; lower absolute values/less negative/indicate more impaired right ventricular function).

**Figure 3:**
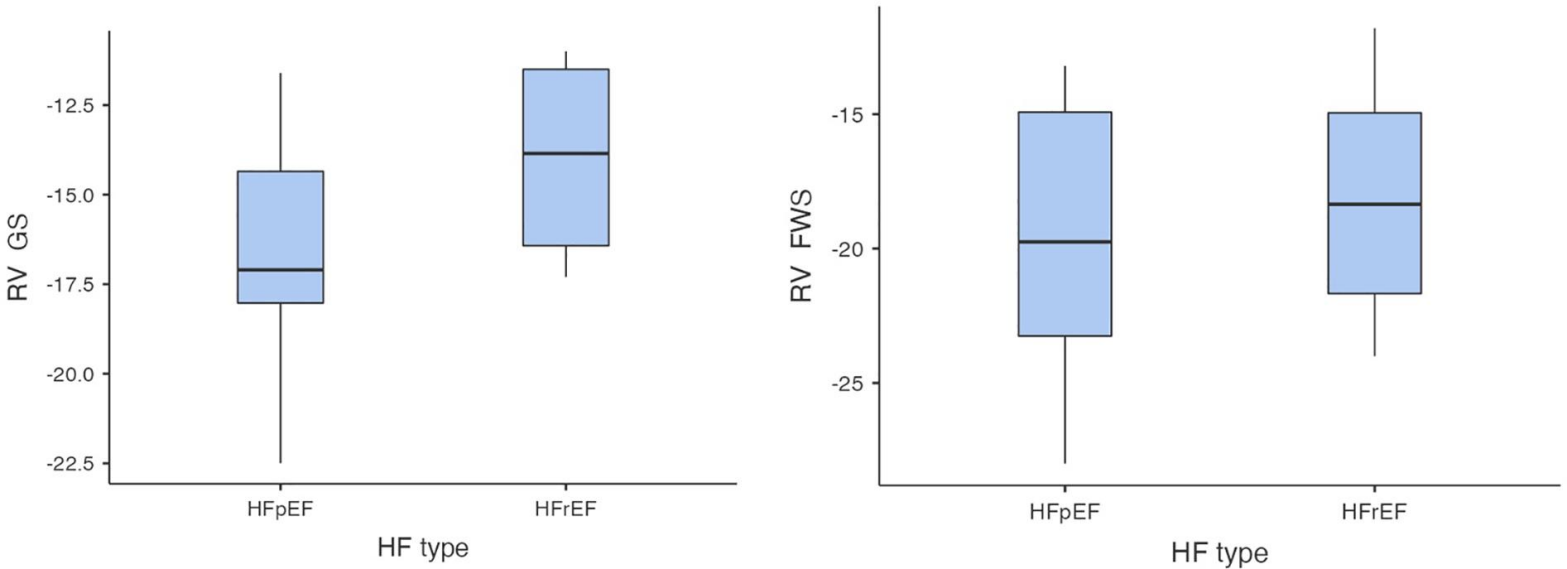
Comparison of RV-GS and RV-FWS between HFrEF and HFpEF subgroups (RV-GS, right ventricular global strain, RV-FWS, right ventricular free wall strain; HF, heart failure; HFrEF, heart failure with reduced left ventricular ejection fraction; HFpEF, heart failure with preserved left ventricular ejection fraction; lower absolute values/less negative/indicate more impaired right ventricular function).

In the first step of analysis, the values of RV-GS and RV-FWS obtained via 2D ST were evaluated using a Student *t*-test statistical analysis, which led to the following results: the values of RV-GS and RV-FWS were significantly lower in both the HFrEF and HFpEF subgroups in comparison with the control group (RV-GS: −15.7 ± 3.32% vs. −22.6 ± 2.26%, *p* < 0.001; RV-FWS: −19.2 ± 4.7% vs. −25.9 ± 2.54%, *p* < 0.001). However, there was neither a significant difference in RV-GS (−14.0 ± 2.82% vs. −16.6 ± 3.32%) nor in RV-FWS (−18.2 ± 4.76% vs. −19.7 ± 4.97%) between the HFrEF and HFpEF subgroups (Figures 2, 3).

There was no statistically significant difference in RV-GS and RV-FWS between the patients, who suffered an adverse event and those without an adverse event in the following 3 and 6 months after index hospitalisation based on our analysis, although patients with RV-GS and RV-FWS greater (more positive) than −20% tended to be at a higher risk of an adverse event in the 6-month follow-up period (1.5-fold higher for RV-GS and 2.5-fold higher for RV-FWS, *p* = 0.07).

### 3.3 Right atrial 2D ST analysis

**Figure 4:**
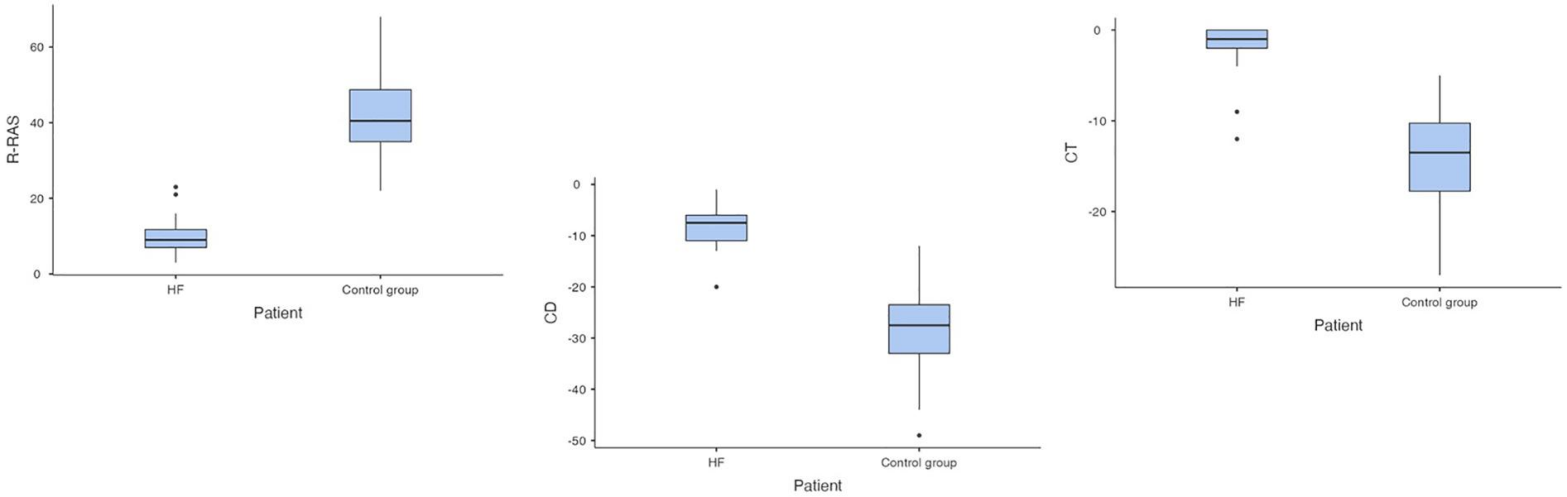
Comparison of right atrial strain in patients with acute decompensated heart failure and controls (HF, heart failure; RAS, right atrial strain; R-RAS, reservoir right atrial strain; CD-RAS, conduit right atrial strain; CT-RAS, contraction right atrial strain).

In the second step of 2D ST analysis, the values of R-RAS, CD-RAS and CT-RAS were analysed. Compared to controls, in ADHF patients significant differences in R-RAS (10.1 ± 5.5% in ADHF vs. 42.5 ± 11.8% in controls, *p* < 0.001), CD-RAS (−8.1 ± 4.5% in patients vs. −27.5 ± 9.3% in controls, *p* < 0.001) and CT-RAS (−2.1 ± 1.0% in ADHF vs. −14.3 ± 6.1% in controls, *p* < 0.001) were found (Figure 4). Comparing ADHF patients with HFrEF and HFpEF (Table 2), no significant differences were found in these parameters (R-RAS: 9.3 ± 3.7% in HFrEF vs. 10.5 ± 6.3% in HFpEF, *p* = 0.88; CD-RAS: −7.0 ± 6.6% in HFrEF vs. −8.7 ± 5.3% in HFpEF, *p* = 0.48; CT-RAS: −2.5 ± 1.0 in HFrEF vs. −1.8 ± 1.0% in HFpEF, *p* = 0.84).

Regarding the association between RAS and adverse events, over the 6-month follow-up period, significant differences in R-RAS (7.1 ± 2.5% vs. 13.9 ± 6.0%, *p* < 0.01) and CD-RAS (−6.1 ± 2.6% vs. −10.6 ± 5.3%, *p* < 0.05) were found in patients with an adverse event, while no differences in CT-RAS (−1.0 ± 0.5% vs. −3.4 ± 1.0%, *p* = 0.43) were present (Table 4).

**Table 4:** Right atrial strain in patients with HFrEF and HFpEF and in patients with and without an adverse event during a 6 month of follow up.

Parameter	HFrEF (6 patients)	HFpEF (12 patients)	Significance (*p* value)
R-RAS (%)	9.33 ± 3.7	10.5 ± 6.3	0.88
CD-RAS (%)	−7.0 ± 6.6	−8.7 ± 5.3	0.48
CT-RAS (%)	−2.5 ± 1.0	−1.8 ± 1.0	0.84
Parameter	HF-related AE (8 patients)	No HF-related AE (10 patients)	Significance (*p* value)
R-RAS (%)	7.1 ± 2.5	13.9 ± 6.0	<0.01
CD-RAS (%)	−6.1 ± 2.6	−10.6 ± 5.3	<0.05
CT-RAS (%)	−1.0 ± 0.5	−3.4 ± 1.0	0.43

AE, adverse event; CD-RAS, conduit right atrial strain; CT-RAS, contraction right atrial strain; R-RAS, reservoir right atrial strain; HFrEF, heart failure with reduced left ventricular ejection fraction; HFpEF, heart failure with preserved left ventricular ejection fraction.

## 4 Discussion

As mentioned, there are limited data regarding the role of RV and RA strain in patients with ADHF. In our study, there were significant differences in RV-GS, RV-FWS, as well as in R-RAS, CD-RAS and CT-RAS in patients with ADHF compared to controls; with no differences detected between patients with HFrEF and HFpEF. This finding implicates that right ventricular and right atrial dysfunction is commonly present in those patients, who develop acute decompensation of HF, with no differences between HFrEF and HFpEF phenotypes. Moreover, in patients who experienced an adverse event in the 6-month period of clinical follow-up, significant differences in R-RAS and CD-RAS were found. Although there were no significant differences in RV-GS and RV-FWS when comparing patients with and without an adverse event, the patients with RV-GS and RV-FWS greater (more positive) than −20% tended to be at a higher risk of these events.

Comparing our data with data from previous studies, for RV strain (RV-GS or RV-FWS), several previously published studies already reported its association with HF-related cardiac events in patients with ADHF (3, 4), with future adverse events in patients with right-ventricular arrhythmogenic cardiomyopathy (6), and it seems that RV-strain can also predict impaired long-term recovery after the first event of decompensation in *de-novo* HFrEF (5), or the degree of hepatic dysfunction in patients with acute worsening of chronic HF (13); suggesting that RV strain (mostly RV-FWS) can be used as a predictor of worse clinical outcome in patients with ADHF, which is in line with our observation.

Furthermore, data regarding the prognostic value of RAS in ADHF are even more limited. As mentioned, several previous studies examined the role of RV strain in patients with HF, the most probable clinical impact of our study is the fact, that this study focuses on the role of the RAS assessment—especially on the changes of CD-RAS and CT-RAS which were not assessed in previously published studies. Although impaired R-RAS has been associated with a worse prognosis in paediatric patients with pulmonary hypertension (7), with kidney dysfunction in patients with tricuspid regurgitation and ADHF (8, 9), and predicted HF development in patients with arrhythmogenic right ventricular cardiomyopathy (14); in fact, apart from our observations of reduced R-RAS in patients with ADHF (which was significantly more impaired in those experiencing an adverse event in the 6-month follow-up period), there is only one other study examining this issue. In this study, Nagai et al. (15) demonstrated in a sample of 226 patients with HF that patients with impaired R-RAS had a significantly higher rate of adverse events, and that R-RAS was an independent predictor of these adverse events. In this study, the prognostic value of CD-RAS and CT-RAS was not examined. Expanding this issue further, our study found also significant differences in CD-RAS when comparing patients, who developed an adverse event in the 6-month follow-up period with those who remained free of these events. These observations suggest the possible role of right atrial dysfunction for predicting un-favourable course of HF. Nevertheless, as the data available so far are only limited to two observational studies, further research will be certainly required. If our results will be confirmed in future trials, the assessment of RAS could possibly allow personalised risk stratification in patients with ADHF.

In addition, our study directly compared the RA and RV strain in patients with ADHF and healthy controls, firstly showing the real changes in RA and RV strain in ADHF compared to “normal values”. Although the results of this confirmation should be interpreted with caution, and limitations listed below should be considered when these results are interpreted, our study showed significant reduction in RAS and RV strain in the settings of ADHF, irrespectively of HF phenotype (HFrEF/HFpEF), suggesting that early changes of these parameters could predict the development/deterioration of HF in future. Nevertheless, this hypothesis was not tested in our study, nor in other previous studies, and therefore this is another issue which needs to be clarified in future research. Therefore, there is no definite answer to the question what is the real clinical implication of our observation. One could speculate that this observation could lead to more personalised approach for ADHF patients, and maybe, in future, the assessment of RAS could identify patients with the risk of HF (HFrEF/HFpEF) development or the risk of HF deterioration in those patients who previously presented with stable clinical state. Summarizing, based on the pilot nature of our study, there is no place for final conclusions, but there is a need for future clinical research on the role of RAS assessment in patients with the risk of HF and on its prognostic role in patients with known developed HF.

## 5 Limitations

With regard to our results, one must consider that there were important limitations to be taken into account when the results of our study are interpreted. First of all, it is recognised that the sample size of the study is relatively small, which is partly due to the fact that 2D ST analysis of RA and RV requires precise measurements and suitable images from the A4C projection, and on many occasions even a modified A4C projection or RV-focused apical four-chamber view, which all depend on the patient's habitus, compliance, degree of dyspnoea, tolerance of horizontal position and other factors as well. Therefore, only those patients, in whom echocardiography allowed us to obtain adequate images for 2D ST analysis were enrolled in our study. This inevitably might have led to selection bias. Therefore, it could be argued that our results are only applicable to acute HF patients with good echocardiographic imaging quality for 2D ST analysis and likely cannot be generalized to all ADHF patients. Second of all, the measurements were not all taken by one cardiologist (but by two individuals), so naturally, there is room for inter-individual variability in recording and interpreting the echocardiographic findings. However, the risk of inter-individual variability was mitigated by having only one individual perform the 2D ST analysis. Third of all, the control group and the HF groups differed in terms of age, and a possibility that control individuals would develop HF in the future cannot be ruled out. Unfortunately, it would be extremely difficult to obtain age-matched control group of healthy individuals due to the increased prevalence of HFpEF-related diseases with higher age. These HFpEF-related diseases (arterial hypertension, atrial fibrillation, obesity and type 2 diabetes, valvular diseases such as aortic stenosis) could, in theory, affect RA and RV strain. As we wanted to demonstrate the true differences in RA and RV strain in patients with acute decompensated HF compared with the sample of truly healthy individuals, we needed to accept this limitation. Finally, the discretion regarding the choice and optimisation of medical therapy for acute decompensated heart failure was left to the attending physician. Actually, there were differences in heart failure pharmacotherapy at admission and upon release between patients with HFrEF and HFpEF. These differences in guideline-recommended therapy were in line with treatment differences for HFrEF and HFpEF, which were recommended at the time of study period, and were also caused by the country's drug administration policy, which was valid at the time of the study (e.g., SGLT-2 inhibitors could not to be administered in patients with HFpEF at that time). All these facts should be taken into consideration when interpreting the results of our study.

## 6 Conclusion

Our study demonstrated a significant difference in RV-GS, RV-FWS, R-RAS, CD-RAS and CT-RAS in patients with ADHF in comparison with the control group; with no evident differences between the HFrEF and HFpEF subgroups. Moreover, significant differences in R-RAS and CD-RAS were demonstrated in patients who developed a HF-related adverse event in the 6-month follow-up period. These results advocate further research of RV and RA strain in patients with ADHF.

## Data Availability

The raw data supporting the conclusions of this article will be made available by the authors, without undue reservation.
